# A Novel Fibrinolytic Protein From *Pheretima vulgaris*: Purification, Identification, Antithrombotic Evaluation, and Mechanisms Investigation

**DOI:** 10.3389/fmolb.2021.772419

**Published:** 2022-01-24

**Authors:** Hai Liu, Jianqiong Yang, Yamei Li, Yunnan Ma, Wenjie Wang, Wanling Zhong, Pengyue Li, Shouying Du

**Affiliations:** ^1^ School of Chinese Materia Medica, Beijing University of Chinese Medicine, Beijing, China; ^2^ College of Pharmacy, Gannan Medical University, Ganzhou, China; ^3^ The First Clinical Medical College, Gannan Medical University, Ganzhou, China

**Keywords:** Pheretima vulgaris, purification, fibrinolytic protein, antithrombotic evaluation, *de novo* sequencing, molecular docking

## Abstract

Thrombotic diseases have been considered major causes of death around the world. Treatments with thrombolytic drugs, such as recombinant tissue-plasminogen activator, urokinase, and streptokinase, are reported to have a life-threatening bleeding tendency. On the contrary, lumbrokinase, identified from *Lumbricus rubellus*, is specific to fibrin and does not cause excessive bleeding. It possesses fibrinolytic activity and activation of plasminogen to dissolve fibrin. Hence, the purification of fibrinolytic protein monomer from earthworm and antithrombotic evaluation and investigation of mechanisms are needed. In this study, a novel fibrinolytic protein EPF3, with strong fibrinolytic activity, was purified from *Pheretima vulgaris* by ion exchange and size exclusion chromatography. SDS PAGE, bottom-up proteomics analysis, *de novo* sequencing, and circular dichroism (CD) analysis were carried out for identification and characterization of it. EPF3, with a molecular weight of 25136.24 Da, consisted of 241 amino acids and contained various forms of secondary structures, including α-helix (3.9%), β-sheet (42.8%), β-turn (21.2%), and random coil (32.1%). It was a trypsin-like serine protease and stable at pH 7.0 to 11.0 and below 40°C. EPF3 was confirmed to possess an antithrombotic effect by *ex vivo* clot lysis test and fibrinogen-thrombin time (Fib-TT) assay. The three-dimensional structure of EPF3 was predicted by SWISS-MODEL. Molecular docking analysis predicted that EPF3 could directly interact with antithrombotic target proteins (fibrin, fibrinogen, and plasminogen), which was further confirmed by further studies. The antithrombotic mechanism of EPF3 was clarified to be outstanding direct fibrinolysis, fibrinogenolytic activity, and certain activation of plasminogen. EPF3 possesses the potential to be developed into a promising antithrombotic agent.

## 1 Introduction

Thrombotic diseases, including cerebral ischemia, acute arterial thrombosis, myocardial infarction, and venous thrombosis, have been considered major causes of death around the world (Patsouras and Vlachoyiannopoulos, 2019). At present, some thrombolytic drugs, such as recombinant tissue-plasminogen activator (rt-PA), urokinase (uPA), and streptokinase (SK), have been widely used to treat thrombosis disorders. However, their side effects, such as heavy hemorrhagic complications, allergic reactions, anaphylaxis, and immunoreactions, are often reported, low specificity for fibrin and relatively high cost are also their deficiencies in clinical application (Vernooij et al., 2009; Wu et al., 2020). As a vicarious therapy, traditional medicinal animals such as earthworms, snakes, and leeches have attracted more attention in the past few decades (Wu et al., 2020).

The earthworms have been traditionally used to treat thrombosis disorders for hundreds of years in China, Indonesia, Japan, and other East Asian countries (Fu et al., 2013). Lumbrokinase, identified from *Lumbricus rubellus* by Mihara et al. (1991), possesses fibrinolytic activity and activation of plasminogen to dissolve fibrin (Wang et al., 2004). It is specific to fibrin and does not cause excessive bleeding. Earthworm fibrinolytic proteins were mainly extracted from *Lumbricus rubellus*, *Eisenia fetida*, and *Pheretima* sp. in the former research (Trisina et al., 2011; Yang et al., 2019). *Pheretima vulgaris* Chen (*P. vulgaris*), widely used in China, is a species of earthworm in the Chinese pharmacopoeia and has been rarely studied. Hence, the purification of active protein monomer, and antithrombotic evaluation and mechanisms investigation are needed. In our former research, we have constructed a local *P. vulgaris* database based on the transcriptome results of *P. vulgaris*.

In this study, a novel fibrinolytic protein EPF3, with five times stronger specific activity than lumbrokinase, was purified from *P. vulgaris* and identified by bottom-up proteomic analysis and *de novo* sequencing. EPF3 was confirmed to possess an antithrombotic effect by *ex vivo* clot lysis test and Fib-TT assay. The antithrombotic mechanisms were direct fibrinolysis, fibrinogenolytic activity, and plasminogen activation, consistent with the results of molecular docking studies. EPF3 possesses the potential to be developed into a promising antithrombotic agent.

## 2 Materials and Methods

### 2.1 Materials

Living earthworms (*P. vulgaris*) were provided by Shanghai Dilong Breeding Base and identified by Professor Du Shouying at Beijing University of Chinese Medicine. The crude extract of *P. vulgaris* was obtained by water extraction and alcohol precipitation. Lumbrokinase, fibrinogen, and thrombin were purchased from China National Institutes for Food and Drug Control. BCA Protein Assay Kit was obtained from Solarbio life sciences (China). Human plasminogen, phenylmethane sulfonyl fluoride (PMSF), soybean trypsin inhibitor (SBTI), N--tosyl-l-phenylalanine (TPCK), and pepstatin were purchased from Sigma (United States). Gel filtration low molecular weight marker protein was obtained from Thermo Fisher Scientific (United States). Superdex 75 Increase 10/300 GL pre-packed columns and HiTrap Q HP pre-packed column were purchased from GE life sciences (United States). Other reagents used were of analytical grade.

Sprague–Dawley (SD) rats were purchased from Hunan SLAC Laboratory Animal Co., Ltd. (Changsha, China). All experimental protocols were approved by the Animal Experimentation Committee of the Beijing University of Chinese Medicine (Beijing, China).

### 2.2 Determination of Fibrinolytic Activity and Protein Content

Fibrin-plate assays were used to estimate fibrinolytic activity according to Astrup and Müllertz (Astrup and Mullertz, 1952), with slight modifications. Simply, fibrinogen solution (2 mg/ml, 8 ml) was mixed with agarose solution (9 mg/ml, 10 ml) and thrombin solution (20BP/mL, 0.25 ml). Then, the mixture was added into a petri dish and kept for 1 h at room temperature to solidify. Next, 10 µL of sample solution was carefully added into a well (6 mm) previously punched on the fibrin plate. The plate was incubated for 18 h at 37°C. The fibrinolytic activity was calculated by measuring the clear zone of dissolving fibrin on the plate. The standard control is lumbrokinase.

Protein content was estimated by BCA Protein Assay Kit. Bovine serum albumin (BSA) was used as the standard. The specific activity was calculated as the ratio of the fibrinolytic activity to protein concentration.

### 2.3 Purification of Fibrinolytic Proteins

The purification of the crude extract of *P. vulgaris* was carried out by ion-exchange chromatography (HiTrap Q HP pre-packed column) and size exclusion chromatography (Superdex 75 Increase 10/300 GL pre-packed column). Specifically, the crude extract was dissolved in buffer A (20 mM Tris-HCl buffer, pH 7.5) and passed through a 0.22 μm filter. Then, the liquid sample was loaded into a HiTrap Q HP pre-packed column equilibrated with buffer A and eluted with a linear gradient of NaCl (from 0–0.35 M, pH 7.5). A total of 88 fractions were collected, and the area of the clear lytic zone was measured. The fractions corresponding to major chromatographic peaks with maximum specific activity were pooled and concentrated, and the solvent was replaced with buffer B (20 mM Tris-HCl buffer pH 7.5 containing 0.3 M NaCl) and then used for further purification.

The sample solution with the maximum specific activity was loaded into a Superdex 75 Increase 10/300 GL pre-packed column and then eluted with buffer B. The fractions corresponding to the main elution peak were collected.

### 2.4 Identification of EPF3

The protein pattern of EPF3 was analyzed using 15% SDS PAGE. The protein band was identified by bottom-up proteomics. Data acquired from LC-MS/MS spectra were searched compared with the earthworm database downloaded from UniProtKB on 7th July 2020 and the local *P. vulgaris* database established through the transcriptome results of *P. vulgaris* by our research group. Specifically, the gel piece was cut from SDS PAGE, destained, and then digested overnight by trypsin. The peptides were extracted for nanoLC-MS/MS analysis. The peptide mixture was dissolved in buffer A (0.1% formic acid), loaded into a reverse-phase trap PepMap C18 column (Thermo, 100 μm × 2 cm, nanoViper) connected to the C18 reverse-phase analytical column (Thermo, 10 cm, 75 μm inner diameter, 3 μm resin), eluted with a linear gradient of buffer B (0.1% formic acid and 84% acetonitrile) at 280 nl/min, and then analyzed by Q Exactive mass spectrometer (Thermo Scientific) with peptide recognition mode enabled. The MS/MS spectrum was searched against the UniProtKB database and local *P. vulgaris* database by Proteome Discoverer 2.1.

To obtain the amino acid sequence of EPF3, four different enzymes were used to digest the protein bands and then applied to *de novo* sequencing. To be specific, the gel piece was digested by enzymes for 16 h and the digestion temperature of chymotrypsin was at 25°C while the digestion temperature of endoproteinase Glu-C, pepsin, and elastase was 37°C. The LC-MS/MS data were searched against the local *P. vulgaris* database to obtain the amino acid sequence.

The secondary structure of EPF3 was determined by a circular dichroism (CD) spectroscopy (Verma and Pulicherla, 2017). The CD spectrophotometer was set with the wavelength from 190 to 600 nm, a path length of 1 mm. The concentration of EPF3 was 0.2 mg/ml. Distilled water was used as the blank solvent. CD values were recorded and further analyzed by Dichroweb (http://dichroweb.cryst.bbk.ac.uk) with K2D as an algorithm.

### 2.5 Effects of pH and Temperature on the Fibrinolytic Activity of EPF3

To determine an optimal pH, the fibrinolytic activity of EPF3 was assayed at 37 °C in different pH values ranging from 6.0 to 9.2 (Deng et al., 2018). The pH stability was obtained by incubating EPF3 with buffers of different pH values ranging from 3 to 11 at 4 °C for 24 h (Phan et al., 2011; Cheng et al., 2015) and then measuring the residual fibrinolytic activity at 37°C by fibrin-plate assays.

The optimal temperature of EPF3 was carried out by estimating the fibrinolytic activity at a range of different temperatures from 25 to 65°C (Cheng et al., 2015). The thermostability was evaluated by incubating EPF3 at a range of temperatures from 25 to 65°C for 1 h and then measuring the residual fibrinolytic activity by fibrin-plate assays.

### 2.6 Influence of Inhibitors and Metal Ions on the Fibrinolytic Activity of EPF3

EPF3 was incubated with different inhibitors (PMSF, TPCK, SBTI, pepstatin, and EDTA) (Verma and Pulicherla, 2017; Deng et al., 2018) and metal ions (Mg^2+^, Fe^2+^, Cu^2+^, and Ca^2+^) (Phan et al., 2011; Cheng et al., 2015) at 37°C for 1 h. Then, the residual fibrinolytic activity was measured. The activity of EPF3 without inhibitors and metal ions was considered to be 100%.

### 2.7 Antithrombotic Evaluation

#### 2.7.1 *Ex Vivo* Clot Lysis Test

The thrombolysis effect of EPF3 was estimated using a clot lysis assay modified according to Babaee et al. (2018); Wu et al. (2020). Rats were anesthetized with 2% pentobarbitone (40 mg/kg) by intraperitoneal injection. The whole blood of rats was taken from the abdominal aorta and incubated in a water bath at 37°C for 4 h. The clot strips were obtained and divided into 5 mm segments, which were placed into sterilized tubes and incubated with samples (EPF3, lumbrokinase) for 4 h at 37°C. Weighting of the clots was conducted at different time points (0, 0.5, 1, 2, and 4 h).

#### 2.7 2 Fibrinogen-Thrombin Time Assay

Assays of Fib-TT (Wu et al., 2019) were applied to evaluate the anticoagulation effect of EPF3.

Specifically, samples (EPF3, lumbrokinase) were first mixed with fibrinogen (0.4%) and then incubated with thrombin (2 P/ml) for 2 min. The Fib-TT was measured by an automatic blood coagulation analyzer (Sysmex CA-500, Japan).

### 2.8 Mechanisms of Antithrombotic Effect

#### 2.8.1 Three-Dimensional Protein Structural Homology Modeling and Docking Studies

Considering EPF3 was a novel protein, the three-dimensional structural homology modeling of EPF3 was conducted by SWISS-MODEL (https://swissmodel.expasy.org/) (Waterhouse et al., 2018; Sharma et al., 2021) based on the amino acid sequence.

ZDOCK (Shah et al., 2019), a fast Fourier transform-based protein docking program, was used for performing docking analysis of EPF3 with fibrin (PDB ID: 2Z4E), fibrinogen (PDB ID: 2OYH), and plasminogen (PDB ID: 1QRZ). The modeled structures and docking models were validated by 4 quality-check tools in a SAVES v6.0 online server, including PROCHECK, ERRAT, Verify 3D, and WHATCHECK (Muhammed et al., 2019; Sharma et al., 2021; Wu et al., 2019).

#### 2.8.2 Study on the Fibrinogenolytic Activity

The fibrinogenolytic activity of EPF3 was displayed by zymography. Time and dose correlation of the fibrinogenolytic activity of EPF3 were conducted according to Wu et al. (2020). Specifically, EPF3 (20 μg/ml, final concentration) was incubated with fibrinogen solution (1 mg/ml, final concentration) at 37°C for 240 min and the samples were collected at different time points (5, 10, 20, 30, 60, 120, and 240 min). In the dose correlation assay, EPF3 (5, 10, 20, 40, 80, and 160 μg/ml, final concentration) and fibrinogen solution (1 mg/ml, final concentration) were applied at 37°C for 60 min, and the samples were collected. The collected samples were separated by 12% SDS PAGE and stained by Coomassie Brilliant Blue R-250.

#### 2.8.3 Analysis of Plasminogen Activation

A fibrin-plate assay was used to analyze plasminogen activation of EPF3. Saline, plasminogen, EPF3, and a mixture of EPF3 and plasminogen were spotted on the fibrin plates, which were incubated at 37°C. The zones of lysis on the plate were measured at different time points (3, 6, and 18 h).

### 2.9 Statistical Analysis

Results in this paper were shown as mean ± SD. All statistical analyses were performed by GraphPad Prism 8.0. The multiple comparisons were determined by one-way ANOVA with the aid of Dunnett’s multiple comparison test. *P* value under 0.05 was regarded as a significant difference.

## 3 Results

### 3.1 Purification of Fibrinolytic Proteins

**FIGURE 1:**
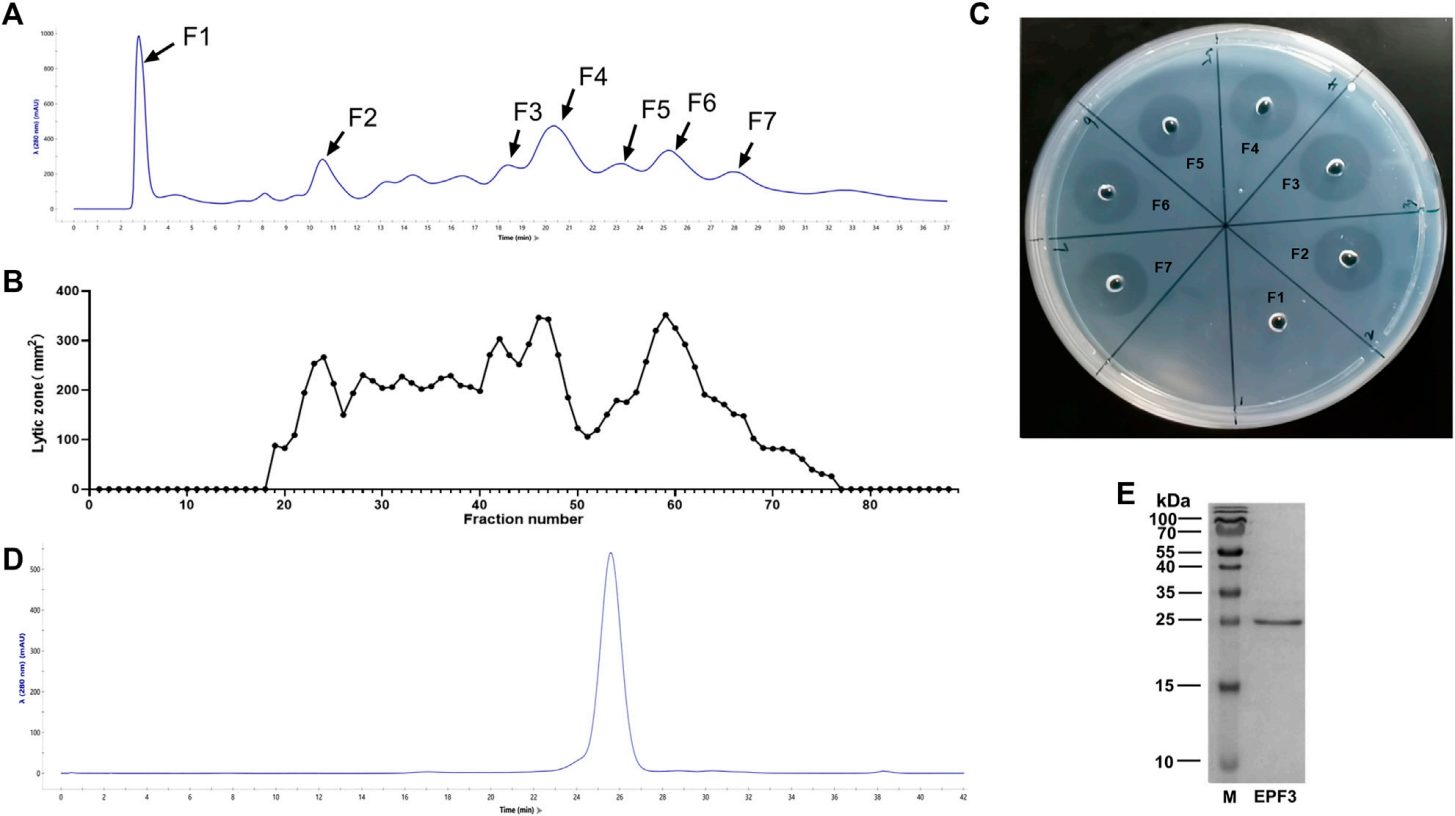
Purification of fibrinolytic proteins. **(A)** Ion-exchange chromatography of *P. vulgaris* crude extract presenting 7 main peaks, the absorbance at 280 nm was shown at the axis. **(B)** The area of the lytic zone of 88 eluted fractions. **(C)** The fibrin-plate assays of 7 main chromatographic peaks. **(D)** Size exclusion chromatography of F3 presenting 1 main peak, the absorbance at 280 nm was shown at the axis. **(E)** SDS-PAGE profiling of EPF3, in which M represents protein marker, and EPF3 appears as a single band.

The crude extract of *P. vulgaris* was loaded into a HiTrap Q HP pre-packed column, and 88 different fractions were collected. The area of the lytic zone of every fraction was measured (Figures 1A,B). The fractions corresponding to 7 major chromatographic peaks were collected, and specific activity was estimated (Figure 1C; Table 1). The fractions of the F3 peak showed the maximum specific activity and were further purified by size exclusion chromatography (Figure 1D). The fractions corresponding to the main elution peak from size exclusion chromatography, named EPF3, were pooled, desalted, concentrated, and lyophilized. The specific activity of EPF3 was 197.1 U/μg, while that of lumbrokinase was 38.3 U/μg. The purity of EPF3 was more than 90%, as estimated by SDS PAGE with Coomassie Brilliant Blue staining (Figure 1E).

**TABLE 1:** The specific activity of fractions corresponding to 7 major chromatographic peaks (mean ± SD, *n* = 3).

Peaks	F1	F2	F3	F4	F5	F6	F7
Specific activity (U/μg)	0.00 ± 0.00	166.56 ± 3.05	181.20 ± 3.97	158.82 ± 6.26	119.83 ± 2.76	158.14 ± 6.43	152.61 ± 1.90

### 3.2 Identification of EPF3

#### 3.2.1 Bottom-Up Proteomic Analysis

Data of EPF3 acquired from LC-MS/MS spectra were searched from the UniProtKB earthworm database and the local *P. vulgaris* database using Proteome Discoverer 2.1. The proteins with high coverage, peptides, and PSMs were acquired from the two databases, respectively (Table 2). By comprehensive consideration of the coverage, peptides, and PSMs, the sequence of EPF3 was speculated to be the same as Ec29745_g1_i1. In order to determine the full sequence of EPF3, further studies were needed.

**TABLE 2:** The identified proteins of EPF3 compared against UniProtKB earthworm database and the local *P. vulgaris* database.

Accession	Database	Coverage	Peptides	PSMs
A0A4S2WI57	UniProtKB earthworm database	10.58	1	1
Ec29745_g1_i1	Local *P. vulgaris* database	23.11	4	33

#### 3.2.2 *De Novo* Sequencing

**FIGURE 2:**
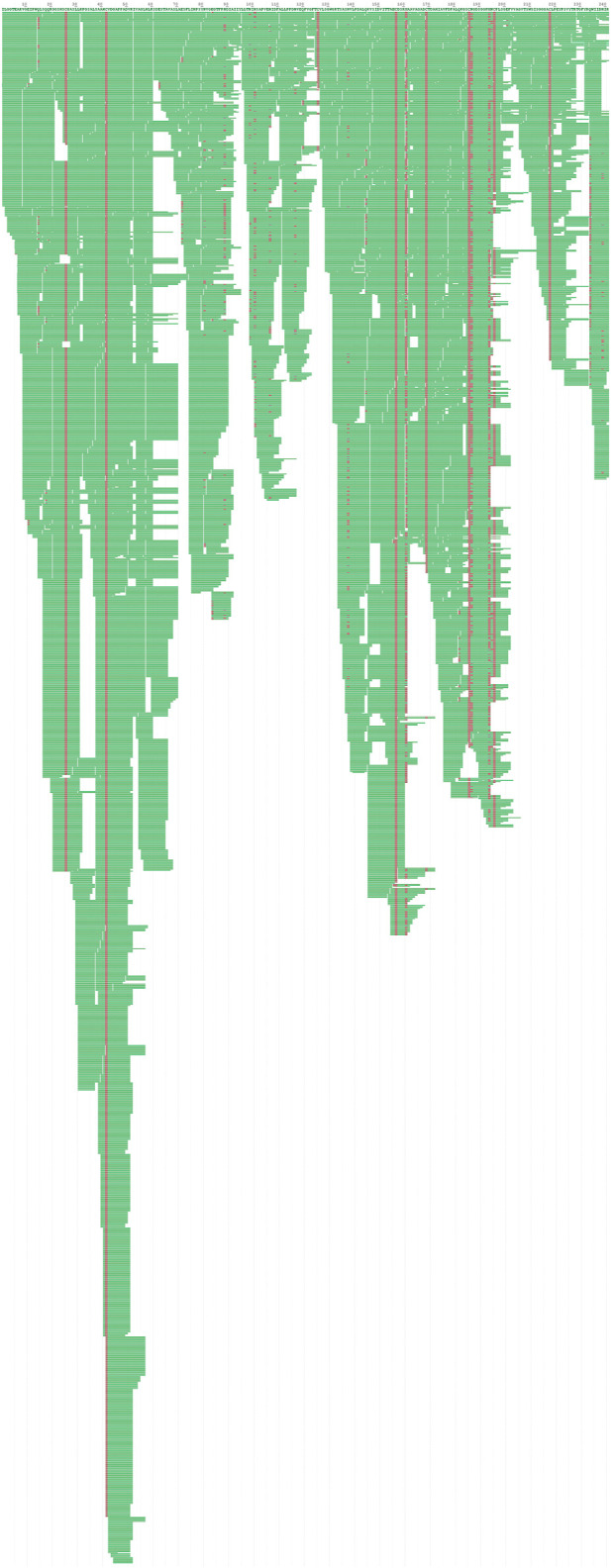
The *de novo* identified sequence and coverage of EPF3.

EPF3 was digested with four different enzymes and then detected by LC-MS/MS. The MS data information was analyzed, and the amino acid sequence of EPF3 was determined against the local *P. vulgaris* database by Byonic software. The Byonic results indicated that the amino acid sequence of EPF3 could cover the full sequence of Ec29745_g1_i1 (Table 3). Peptides digested by enzymes can cover each other (Figure 2). The MW of EPF3 calculated by the ExPASy server (https://web.expasy.org/protparam/) was 25,136.24 Da. Collectively, the amino acid sequence of EPF3 obtained by *de novo* sequencing analysis was highly reliable and shown as follows:

**TABLE 3:** The Byonic identification of EPF3.

Sample	Best sore	Spectra	Peptides	Coverage (%)	Intensity
EPF3	1359.9	10,618	1,460	100	1.47E + 11

ILGGTEARVGEIPWQLSQQRGGSHSCGASLLRPGSALSAAHCVDGAPPADVRIVAGLHLRSDESTAVASLAESFLIHPSYNVGEGTFPNDIAIIYLLTNINSAPVENIDFALLPPDNVEQFVGFTCVLSGWGRTSASNVLPDALQKVSIDVITTAECDSRMAAVAGADCTDAHIAVFDPALQKGSCNGDSGGPMNCPLSGEFVVAGVTSWGISGGGACLPEYPSVYTRTGFYRQWIIDNIR

The sequence of EPF3 was analyzed by BLAST on two online websites (https://blast.ncbi.nlm.nih.gov/Blast.cgi and https://www.uniprot.org/blast/). As the same sequence was not found, EPF3 was considered a novel protein.

#### 3.2.3 The Secondary Structure of EPF3

**FIGURE 3:**
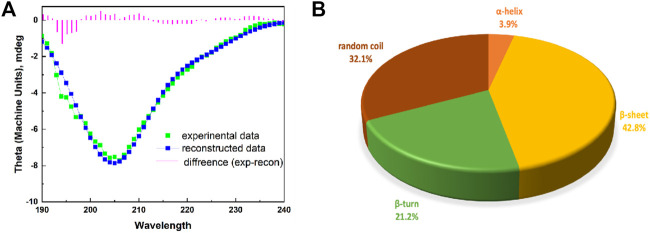
The secondary structure of EPF3. **(A)**: A modeled CD spectrum of EPF3 by Dichroweb online tool with K2D algorithm. The spectra include experimental data (green color), reconstructed data (blue color), and the difference (violet color). **(B)**: A pie graph representing various forms of secondary structure in EPF3.

EPF3 contained various forms of secondary structures, including helix, sheet, turn, and random coil. The ratios of α-helix, β-sheet, β-turn, and random coil were 3.9, 42.8, 21.2, and 32.1%, respectively (Figure 3).

#### 3.2.4 Effects of pH and Temperature

**FIGURE 4:**
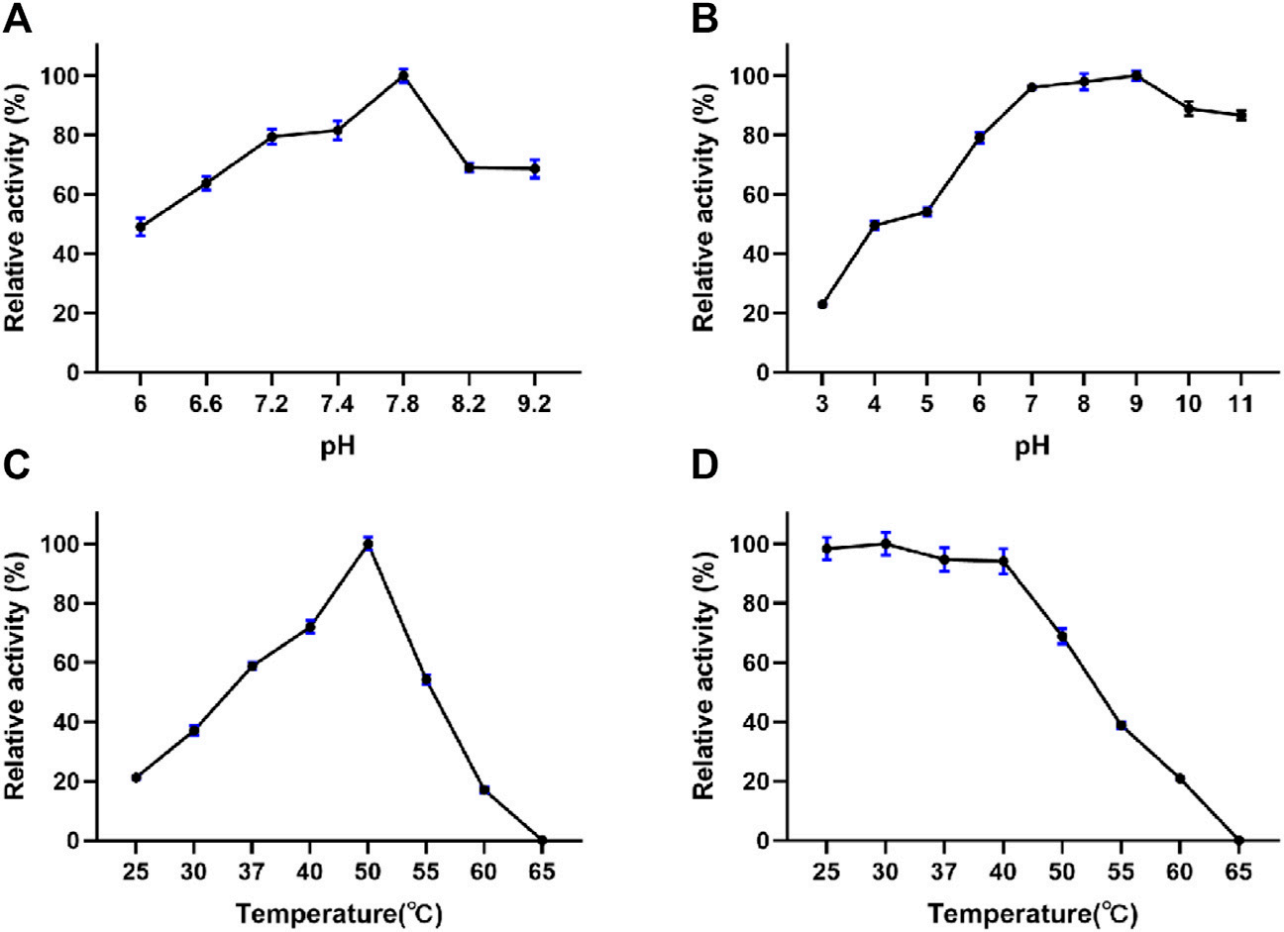
Effects of pH and temperature on the fibrinolytic activity of EPF3 (*n* = 3). **(A)** pH optimum was determined in the pH range of 6.0–9.2. **(B)** pH stability was assessed by measuring the residual activity after incubating EPF3 with buffers of different pH ranges of 3.0–11.0 at 4°C for 24 h. **(C)** Temperature optimum was determined by measuring fibrinolytic activity at a temperature range of 25–65°C. **(D)** Thermal stability was assessed by measuring the residual activity after incubating EPF3 at different temperatures’ range of 25–65°C for 1 h.

The fibrinolytic activity and stability of EPF3 were highly influenced by pH and temperature. The optimal pH of EPF3 was determined to be pH 7.8 (Figure 4A). EPF3 was stable at pH values ranging from 7 to 11, and the fibrinolytic activity was greatly reduced below pH 6.0 (Figure 4B). EPF3 exhibited the maximal fibrinolytic activity at 50°C (Figure 4C), which was an optimal temperature. The result of thermostability showed EPF3 was stable at or below 40°C (Figure 4D). It can be postulated that EPF3 was stable at the pH and temperature in the human body.

#### 3.2.5 Effects of Inhibitors and Metal Ions

The effects of inhibitors and metal ions on EPF3 were estimated by measuring residual fibrinolytic activity after incubation (Table 4). The fibrinolytic activity of EPF3 was absolutely inhibited by PMSF and SBTI, while TPCK, EDTA, Fe^2+^, and Cu^2+^ showed partial inhibition. Pepstatin, Mg^2+^, and Ca^2+^ did not show any inhibitory activity of EPF3. The results suggested that EPF3 might be a trypsin-like serine protease.

**TABLE 4:** Effects of inhibitors and metal ions on fibrinolytic activity against EPF3 (mean ± SD, *n* = 3).

Inhibitors and metal ions	Concentration (mM)	Residual activity (%)
Control	—	100.0 ± 1.33
PMSF	1	0.00 ± 0.00
TPCK	1	63.80 ± 2.57
SBTI	0.1	0.00 ± 0.00
Pepstatin	0.5	104.82 ± 3.73
EDTA	1	81.23 ± 1.55
Mg^2+^	5	101.81 ± 1.66
Fe^2+^	5	83.79 ± 2.86
Cu^2+^	5	60.68 ± 1.81
Ca^2+^	5	98.79 ± 1.29

### 3.3 Antithrombotic Effects

#### 3.3.1 Thrombolysis Effect

**FIGURE 5:**
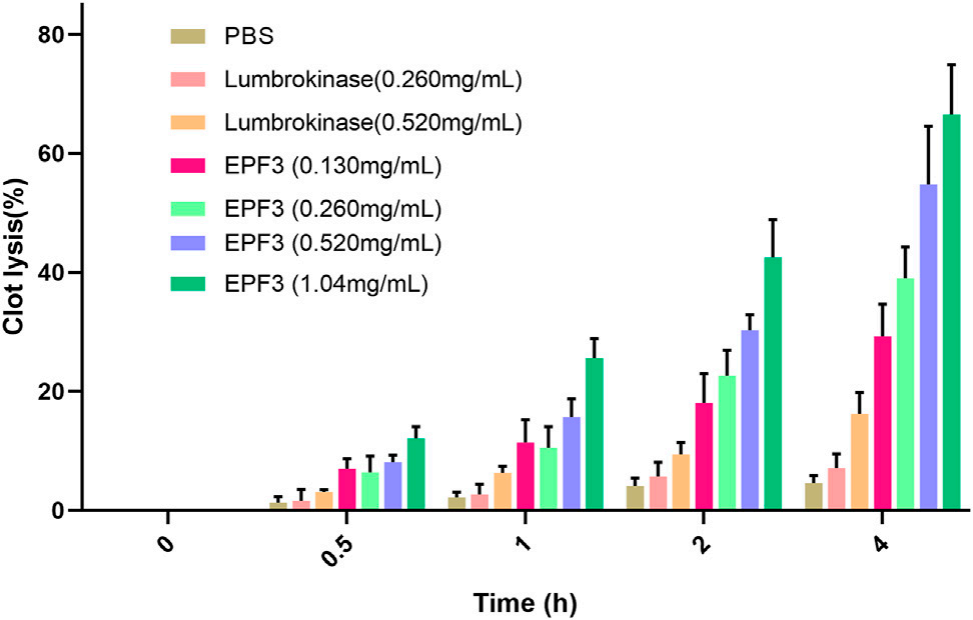
The lysis of blood clot by EPF3 compared with lumbrokinase (*n =* 3).

Clot lysis assay showed that EPF3 possessed a strong ability to hydrolyze clots in a time- and dose-dependent manner (Figure 5). The low (0.130 mg/ml) and high (1.04 mg/ml) concentration of EPF3 could hydrolyze about 29.25 and 66.50% of blood clots after 4 h of incubation, respectively. Lumbrokinase (0.520 mg/ml) showed just a 16.24% thrombolysis rate, while EPF3 at the same concentration displayed 54.80%.

#### 3.3.2 Anticoagulation Effect

**FIGURE 6:**
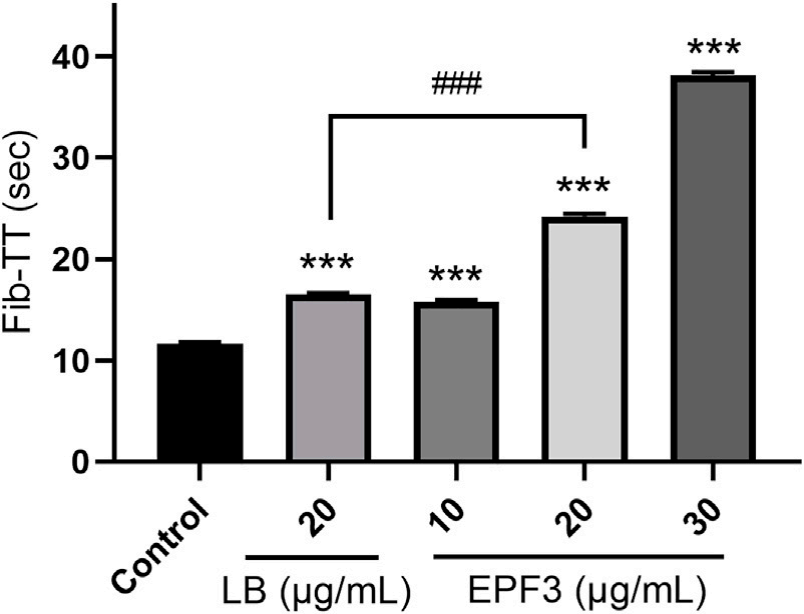
The Fib-TT of EPF3 compared with lumbrokinase (*n* = 3). ***, *p* < 0.0001 compared with control; ^###^ *, p* < 0.0001 EPF3 (20 μg/ml) compared with lumbrokinase (20 μg/ml).

Assays of Fib-TT displayed that EPF3 possessed a strong anticoagulation effect in a dose-dependent manner (Figure 6). EPF3 and lumbrokinase could significantly increase the Fib-TT. The Fib-TT of EPF3 (20 μg/ml) was 24.20 s, while lumbrokinase at the same concentration displayed just 16.53 s.

### 3.4 Antithrombotic Mechanisms

#### 3.4.1 Three-Dimensional Protein Modeled Structure

**FIGURE 7:**
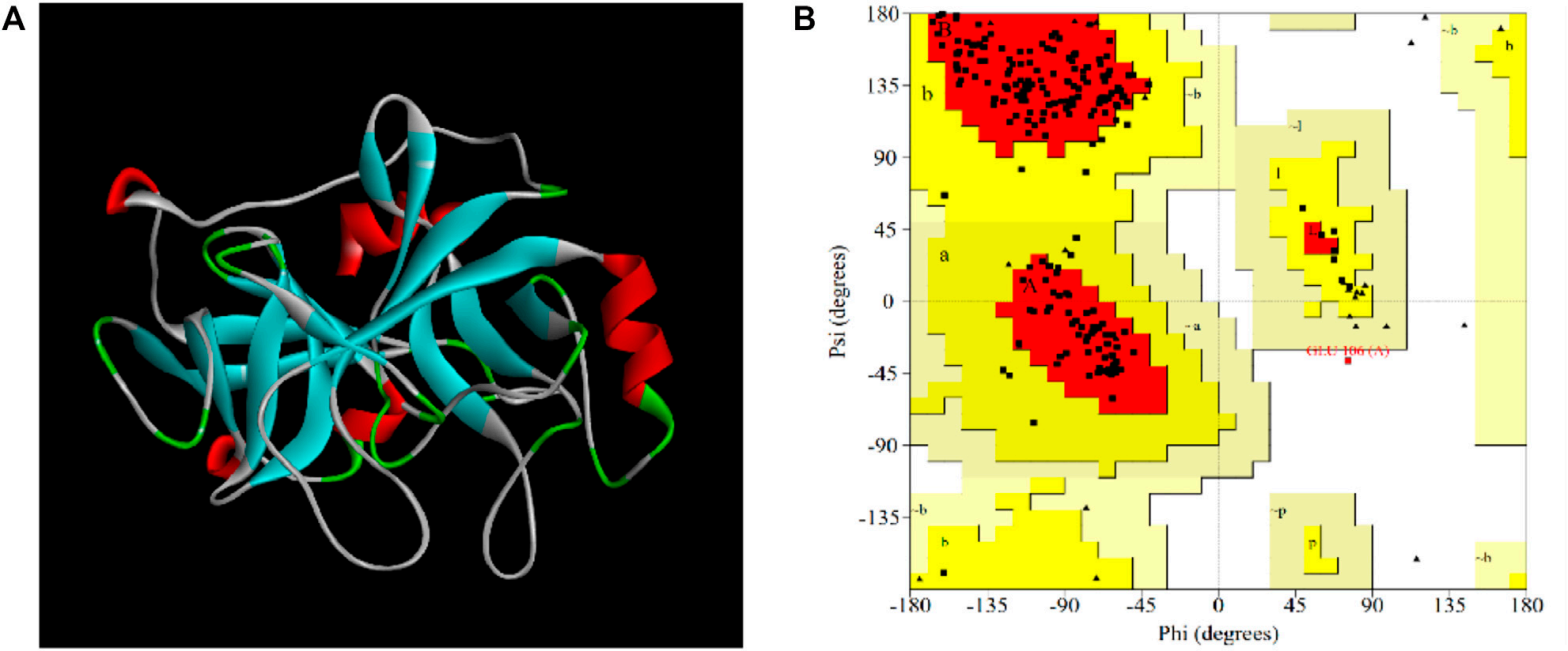
The visual crystal structure **(A)** and Ramachandran plot **(B)** of EPF3 structure model. Residues present in favored, additional allowed, generously allowed, and disallowed regions were shown with red, luminous yellow, green, and white color demarcation, respectively.

In our study, the amino acid sequence of EPF3 was input into the SWISS-MODEL online server. Then, the top 5 proteins with “Seq Identity” greater than 39% were selected as templates for homology modeling. The best model with the highest GMQE (0.85) and QMEANDisCo Local (0.82) was constructed by taking earthworm fibrinolytic enzyme component A from Eisenia fetida (PDB: 1m9u) as a template with 56.78% Seq Identity. The quality of the model was further estimated by SAVES v6.0 online server (Figure 7A). The Ramachandran plot showed 88.3% of residues in most favored regions, 11.2% of residues in additional allowed regions, 0.0% of residues in generously allowed regions, and 0.5% of residues in disallowed regions (Figure 7B). ERRAT (overall quality factor), Verify 3D score, and WHATCHECK of the best model were 99.5, 84.3, and 97.07%, and “Pass,” respectively. Therefore, the best model of EPF3 was proved to be qualified and could be used for further molecular docking analysis.

#### 3.4.2 Molecular Docking Study of EPF3 With Target Proteins

**FIGURE 8:**
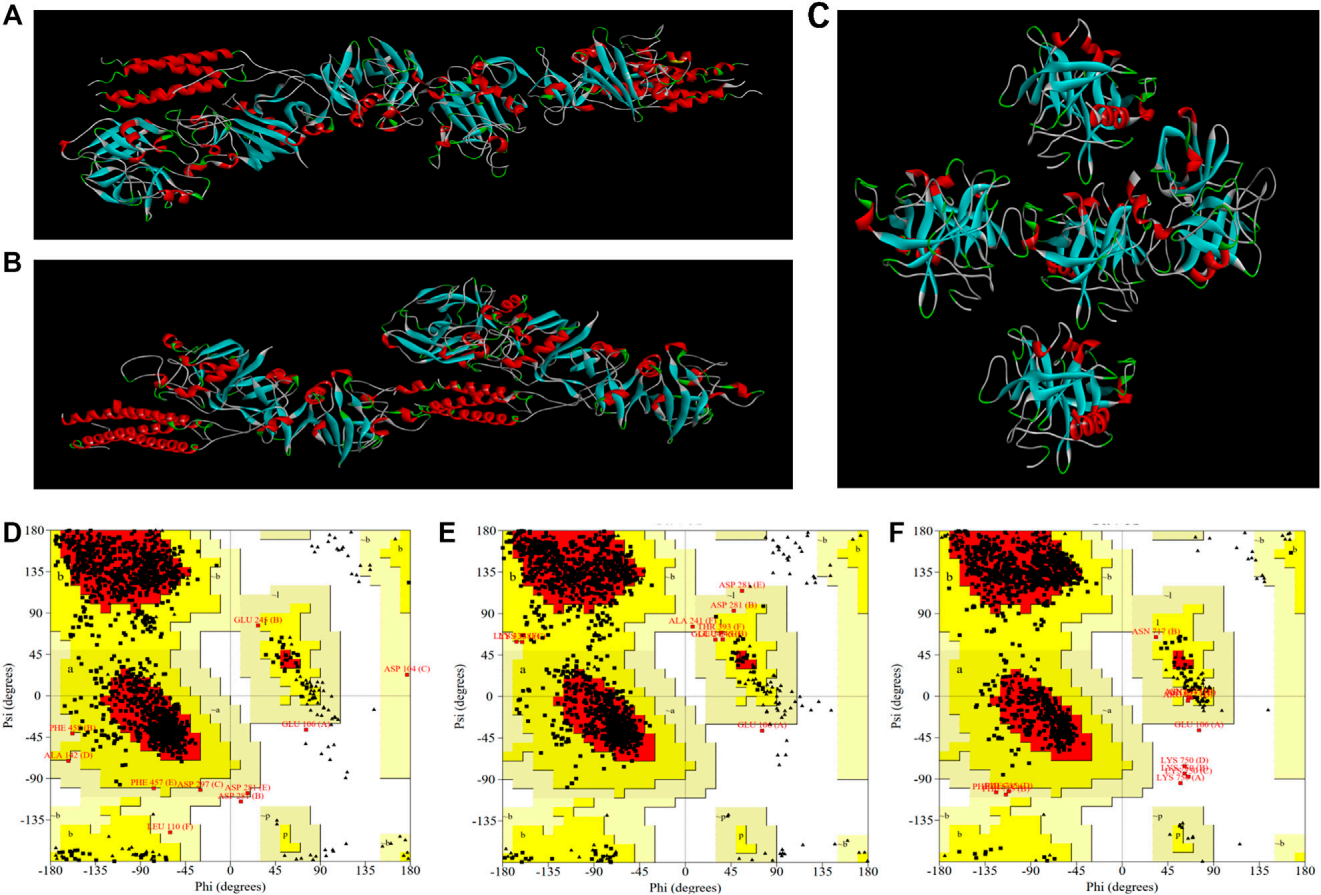
The visualized crystal structures of EPF3 molecular docking model with fibrin **(A)**, fibrinogen **(B),** and plasminogen **(C)**. The Ramachandran plots of EPF3 molecular docking model with fibrin **(D)**, fibrinogen **(E),** and plasminogen **(F)**. Residues presented in favored, additional allowed, generously allowed, and disallowed regions were shown with red, luminous yellow, green, and white color demarcation, respectively.

Molecular docking studies of EPF3 with target proteins (fibrin, fibrinogen, and plasminogen) were conducted by ZDOCK 3.0.2. The model with the highest ZDOCK Score was selected from each molecular docking model (Figures 8A–C) and further estimated by the SAVES v6.0 online server (Table 5). The docking model between EPF3 and fibrinogen exhibited the highest ZDOCK Score, and the Ramachandran plot (Figures 8D–F) revealed that the docking models between EPF3 and fibrin, and fibrinogen were more reasonable, with the 99.3% of the residues in most favored regions and additional allowed regions. The ERRAT score of the docking model between EPF3 and plasminogen was the highest.

**TABLE 5:** The ZDOCK Score and SAVES evaluation of docking models of EPF3.

Ligand	Fibrin (PDB ID: 2Z4E)	Fibrinogen (PDB ID: 2OYH)	Plasminogen (PDB ID: 1QRZ)
ZDOCK Score	1333.232	1518.984	1377.217
MF regions^a^ (%)	82.1	84.2	86.9
AA regions^b^ (%)	17.2	15.1	11.9
GA regions^c^ (%)	0.6	0.6	0.7
DA regions^d^ (%)	0.1	0.1	0.5
ERRAT^e^ (%)	90.18	88.45	86.86
Verify 3D^f^ (%)	80.53	80.44	94.93
WHATCHECK	Pass	Pass	Pass

a Notes. Ramachandran plot: residues in most favored regions.

b Ramachandran plot: residues in additional allowed regions.

c Ramachandran plot: residues in generously allowed regions.

d Ramachandran plot: residues in disallowed regions.

e Overall quality factor generated by ERRAT, server.

f Averaged 3D-1D score ≥ 0.2 generated by Verify 3D server.

Collectively, the results of ZDOCK Score and SAVES evaluation showed that the conformation of the docking model between EPF3 and fibrin, fibrinogen, and plasminogen were reasonable (Muhammed et al., 2019; Shah et al., 2019). We speculated that EPF3 could directly interact with fibrin, fibrinogen, and plasminogen.

#### 3.4.3 Fibrinogenolytic Activity of EPF3

**FIGURE 9:**
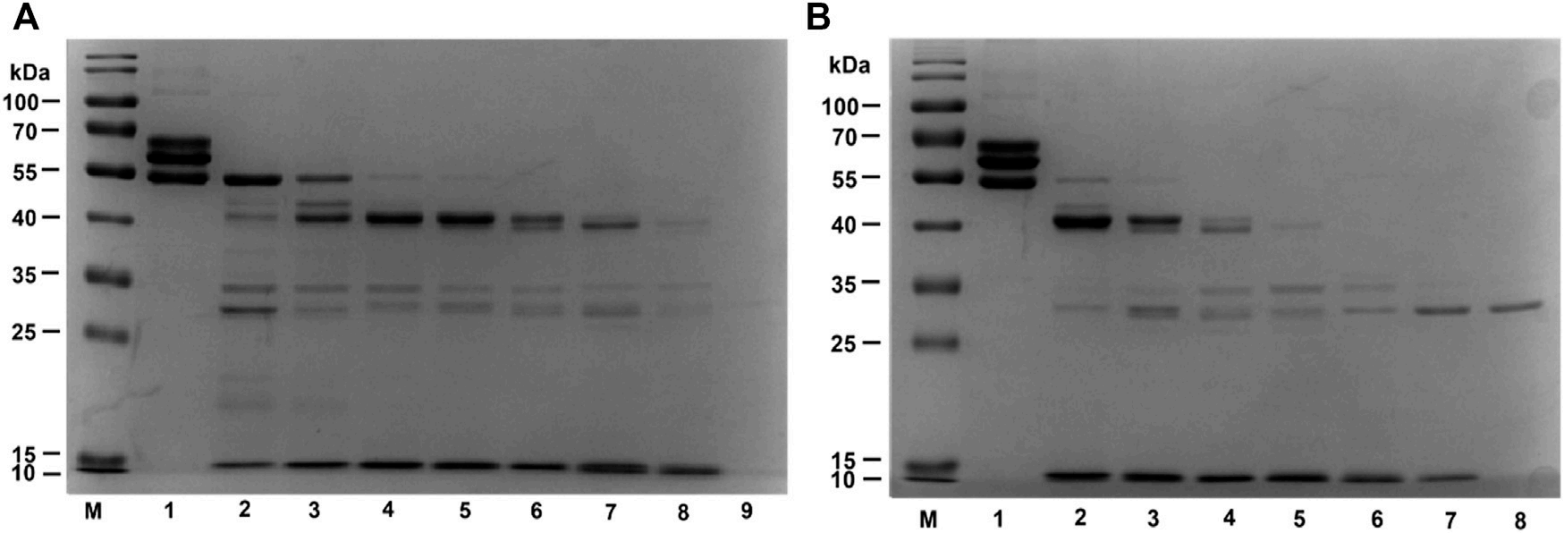
Fibrinogen hydrolysis by EPF3 in a time-dependent manner **(A)** and dose-dependent manner **(B)**. **(A)** Lane 1: fibrinogen; Lane 9: EPF3; Lanes 2 to 8: aliquots were taken at 5, 10, 20, 30, 60, 120, and 240 min, respectively. **(B)** Lane 1: fibrinogen; Lane 8: EPF3; Lanes 2 to 7: different concentrations of EPF3 (5, 10, 20, 40, 80, and 160 μg/ml) reacted with fibrinogen at 37°C for 60 min, respectively.

The hydrolysis pattern of EPF3 on fibrinogen was investigated *via* 12% SDS PAGE. Fibrinogen can be rapidly hydrolyzed by EPF3 in a time- and dose-dependent manner. Specifically, fibrinogen without EPF3 was separated into three chains (α-chain, β-chain, and γ-chain), while fibrinogen was incubated with EPF3, α-chain and β-chain disappeared first, followed by γ-chain, and then, the hydrolyzed fragments were further hydrolyzed into smaller fragments (Figure 9A).

After incubating fibrinogen with various concentrations of EPF3 for 60 min, the α-chain and β-chain of fibrinogen were completely hydrolyzed by EPF3 at low concentration (5 μg/ml), while EPF3 could hydrolyze γ-chain completely at the concentration of 20 μg/ml (Figure 9B).

#### 3.4.4 Plasminogen Activation of EPF3

**FIGURE 10:**
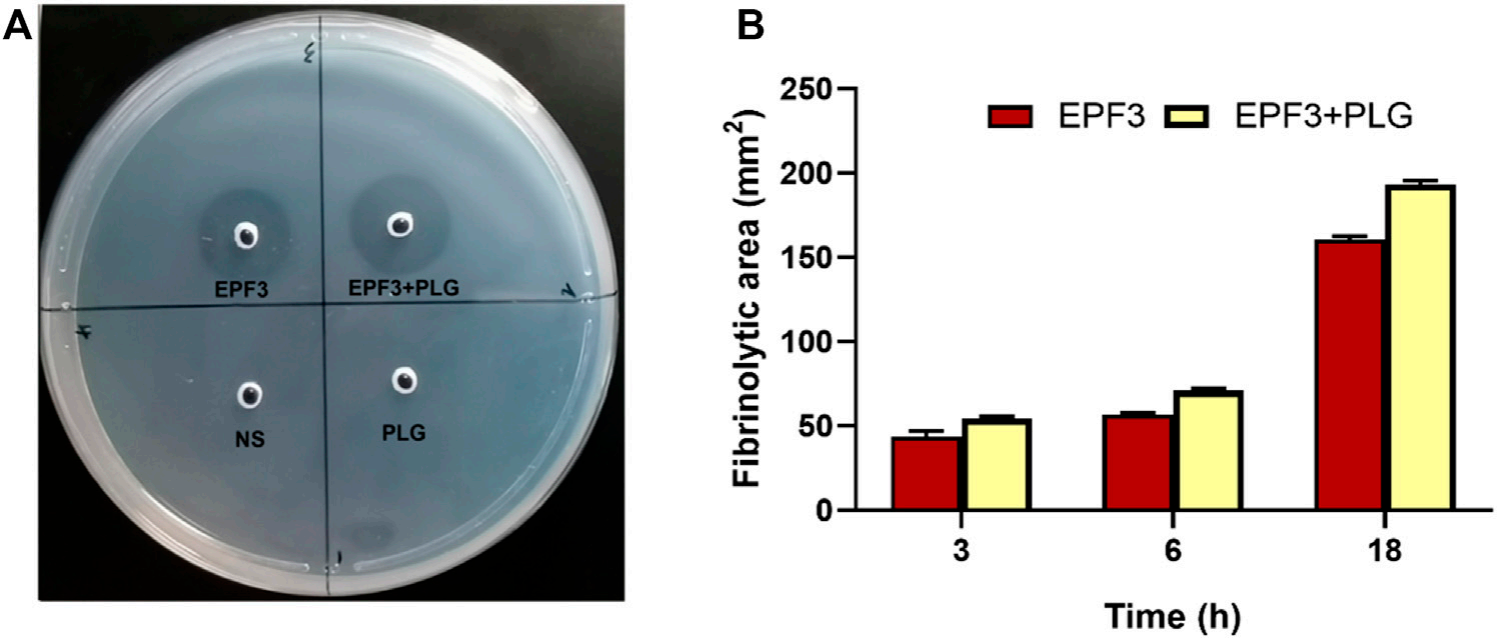
Analysis of fibrinolysis and plasminogen activation by EPF3. **(A)**: fibrin plate. 10 µL sample solutions (Saline, plasminogen, EPF3, a mixture of EPF3, and plasminogen) were placed on the fibrin plates and then incubated at 37°C for 18 h **(B)**: The fibrinolytic area at 3, 6, and 18 h (*n =* 3).

To estimate plasminogen activation of EPF3, the zones of fibrinolysis were measured at different time points. The fibrinolytic zone of the mixture of EPF3 and plasminogen is 20.2% bigger than that of EPF3 (Figure 10). These revealed that EPF3 could partially activate plasminogen to plasmin. In addition, the result also showed EPF3 possessed a direct fibrinolysis activity.

## 4 Discussion

Thrombosis-related diseases have been the leading cause of death worldwide (Patsouras and Vlachoyiannopoulos, 2019). Searching for new effective agents from traditional medicinal animals such as earthworms and snakes and for clinical application has attracted much attention. The earthworms have been used in the treatment of thrombotic diseases for many centuries in East Asian countries (Fu et al., 2013), and the preparations of lumbrokinase have been used in clinical practice in many countries. Nonetheless, *P. vulgaris*, widely used in China, has rarely been studied. Its antithrombotic substance basis and mechanism are still unclear.

Because the antithrombotic proteins isolated from earthworms are mainly fibrinolytic proteins (Mihara et al., 1991; Cho et al., 2004; Trisina et al., 2011), in our study, the crude extract of *P. vulgaris* was isolated and purified based on fibrinolytic activity (Cho et al., 2004). Considering the follow-up experiments of fibrinolytic protein, the fractions corresponding to 7 major chromatographic peaks after ion-exchange chromatography were collected. Then, the fractions with the maximum specific activity were purified by size exclusion chromatography to obtain EPF3. It has five times stronger fibrinolytic activity than lumbrokinase. A relatively large amount of EPF3 can be obtained through this simple process.

The identification of the full sequence of protein remains a challenge. Bottom-up proteomics analysis and the Edman degradation assay were conventional methods (Phan et al., 2011; Verma and Pulicherla, 2017). In this study, the possible sequence of EPF3 was determined by bottom-up proteomics analysis together with the transcriptome. Then, the full sequence of EPF3 was finally identified through *de novo* sequencing. The protein coverage was 100%, which was highly reliable.

It was observed that EPF3 was stable at pH 7.0 to 11.0 and below 40°C with over 80% fibrinolytic activity, which corresponds to the pH and temperature in the human body (Cheng et al., 2015).

Almost all known earthworm fibrinolytic proteins are serine proteases which can be divided into various types such as trypsin-like serine protease, chymotrypsin-like serine protease, and elastase-like protease (Nakajima et al., 2003; Yan et al., 2010; Mander et al., 2011). Studies on the effect of inhibitors and metal ions on enzyme activity are often used to determine types of enzymes. PMSF is known as an effective inhibitor for serine proteases, while SBTI is a specific inhibitor for trypsin (Phan et al., 2011; Deng et al., 2018). The strong inhibition of fibrinolytic activity by PMSF and SBTI indicates that EPF3 is a trypsin-like serine protease. The results, predicted by the CD-search tool in NCBI (https://www.ncbi.nlm.nih.gov/Structure/cdd/wrpsb.cgi) (Marchler-Bauer et al., 2005), revealed that EPF3 belonged to trypsin-like serine proteases. The above two results confirmed each other.

*Ex vivo* clot lysis test and *in vitro* Fib-TT assays showed EPF3 has the activity of thrombolysis and anticoagulation, which confirmed it possessed an antithrombotic activity. Fibrin is the main protein component of human blood clots (Prasad et al., 2006). Generally, direct degradation of fibrin or indirect activation of plasminogen to plasmin might be the underlying mechanisms of fibrinolytic proteins in regulating thrombolysis (Park et al., 2007; Jin et al., 2017). Fibrinogen is a major clotting factor in the coagulation pathway. The decrease in fibrinogen leads to an anticoagulation effect in circulation (Zhao et al., 2007). Therefore, fibrin, fibrinogen, and plasminogen were selected as antithrombotic targets to conduct molecular docking analysis with EPF3. The results showed that EPF3 could directly interact with antithrombotic above three target proteins.

Fibrinogen contains three chains (α-chain, β-chain, and γ-chain). The α-chain is considered to play a key role in fibrin formation induced by thrombin (Zhao et al., 2007; Li et al., 2021) while the γ-chain is the main site of fibrinogen for interaction with platelet (Hawiger et al., 1982). A fibrinolytic protein purified from *Staphylococcus* sp. strain AJ is able to degrade only α-chain of fibrinogen (Choi et al., 2009), while other fibrinolytic enzymes isolated from *Bacillus* sp. nov. SK006 can degrade only β-chain (Hua et al., 2008). In addition, blood viscosity is directly related to fibrinogen level (Ozcan Cetin et al., 2019). EPF3 could degrade three chains of fibrinogen and further hydrolyzed hydrolysates into smaller fragments, which may be the mechanism of its anticoagulation. In the meantime, our studies revealed that EPF3 possessed activities of direct fibrinolysis and plasminogen activation, which are most likely to be the mechanism of its powerful thrombolytic ability.

## 5 Conclusion

In brief, a novel fibrinolytic protein named EPF3 was purified from *P. vulgaris* by column chromatography, and the full sequence was identified by *de novo* sequencing. EPF3 was confirmed to possess an antithrombotic effect, which was based on its activity of thrombolysis and anticoagulation. Further studies clarified that the mechanism was fibrinogen hydrolysis, direct fibrinolysis, and plasminogen activation. Therefore, EPF3 possesses the potential to be developed into a promising antithrombotic agent for treating or preventing thrombus-related diseases. In addition, the antithrombotic effect *in vivo*, subsequent heterologous expression, and enzyme active sites of EPF3 are necessary to be further investigated.

## Data Availability

The datasets presented in this study can be found in online repositories. The names of the repository/repositories and accession number(s) can be found in the article/Supplementary Material.
