# Supplementary material for: A Novel Fibrinolytic Protein From Pheretima vulgaris: Purification, Identification, Antithrombotic Evaluation, and Mechanisms Investigation
Source: Front Mol Biosci. 2022 Jan 24;8:772419. doi: 10.3389/fmolb.2021.772419 (PMC8819685; doi:10.3389/fmolb.2021.772419)
Supplement: Supplementary file 1 [file Table1.pdf]

Supplementary Material

Supplementary Table 1 the amino acid sequence of EPF3.

Protein name	the amino acid sequence
EPF3	ILGGTEARVGEIPWQLSQQRGGSHSCGASLLRPGSALSAA HCVDGAPPADVRIVAGLHLRSDESTAVASLAESFLIHPSYN VGEGTFPNDIAIIYLLTNINSAPVENIDFALLPPDNVEQFVGF TCVLSGWGRTSASNVLPDALQKVSIDVITTAECDSRMAAV AGADCTDAHIAVFDPALQKGSCNGDSGGPMNCPLSGEFVV AGVTSWGISGGGACLPEYPSVYTRTGFYRQWIIDNIR
